# Grey Relational Analysis Combined With Network Pharmacology to Identify Antioxidant Components and Uncover Its Mechanism From Moutan Cortex

**DOI:** 10.3389/fphar.2021.748501

**Published:** 2021-10-07

**Authors:** Yingchun Zhang, Xiaoyi Wu, Xinhui Wang, Yue Zeng, Yixuan Liao, Ruizhi Zhang, Fuqiang Zhai, Zhongliang Zeng

**Affiliations:** ^1^ College of Pharmaceutical Sciences and Chinese Medicine, Southwest University, Chongqing, China; ^2^ School of Traditional Chinese Medicine, Capital Medical University, Beijing, China; ^3^ Chongqing University of Arts and Sciences, Chongqing, China

**Keywords:** antioxidant, grey relational analysis, network pharmacology, paeonol, paeonia suffruticosa andr

## Abstract

The present study determines the potential antioxidants in Moutan Cortex (MC) and predicts its targets of anti-oxidative activities. The quantitative analysis and the free radical scavenging assays were conducted to detect the main components in MC and assess its anti-oxidant activities. The grey relational analysis and the network pharmacology approach were employed to predict its key components and targets of anti-oxidant activities. Six main constitutes in MCs were quantified by high performance liquid chromatography (HPLC) and its anti-oxidant activities were evaluated by DPPH and ABTS free radical scavenging methods. Then grey relational analysis was employed to predict the key components acting on anti-oxidative activity based on the chem-bio results. The predicted components and its mechanisms on anti-oxidation were uncovered by network pharmacology approach and cell test, respectively. The content of paeonol and paeoniflorin accounts for more than 80% the whole content of detected components. However, the two main ingredients showed a great variety among MCs. The antioxidant capacities of MCs also showed a great discrepancy based on DPPH and ABTS methods. The key components acting on anti-oxidation were identified to be paeonol, gallic acid and benzoylpaeoniflorin, and their potential therapeutic targets were predicted and verified, respectively. The present results reveal that MC has a significant antioxidant activity and the compounds of paeonol, gallic acid and benzoylpaeoniflorin could be considered as the promising antioxidant candidates with the property of suppressing oxidative stress and apoptosis.

## Introduction

Free radicals play an important role in maintaining homeostasis at the cellular level in the normal healthy tissues. However, when the body are exposed to the different physicochemical conditions, more free radicals such as reactive oxygen species (ROS) and reactive nitrogen species (RNS) will be generated and thus disturb the balance of ROS generation and antioxidant defense systems, generally resulting in the oxidative stress (Devasagayam et al., 2004) and subsequently causing the damage of cell membrane, protein and DNA (Nijhawana et al., 2019). The current investigations have found the close relationship between oxidative stress and human disease. More and more evidence reveals that the oxidative stress caused by ROS participates in the development of aging, cancer (Kudryavtseva et al., 2016), neurodegenerative diseases (Chauhan et al., 2006), cardiovascular and metabolic diseases (Incalza et al., 2017), and psychiatric disorders (Newton et al., 2015). Antioxidants from nature such as vitamins, flavonoids and phenolic acid can effectively counter oxidative stress by scavenging free radicals (Halliwell et al., 1992). Therefore, the exogenous antioxidants from food or supplements are required to balance the ROS to normal levels in biological systems under oxidative stress status. In recent years, the increasing numbers of natural antioxidants are continuously found in medicinal plants (Akwu et al., 2019; Hamed et al., 2019; Makinde et al., 2019). Thus, the medicinal plants with potent anti-oxidative activity play an important role in prevention and treatment of diseases related to oxidative stress.

*Paeonia suffruticosa* Andr, belonging to Paeoniaceae family, is a deciduous shrub with nearly worldwide distribution. The dried bark of *Paeonia suffruticosa* Andr, commonly called Moutan Cortex (MC), has been used in China for a long history. Many studies have identified and reported more than one hundred of ingredients from MC, including phenols, monoterpenes, monoterpene glycosides, flavonoids, tannins, and triterpenoids (Wang et al., 2019). Recent studies have revealed that MC has strong pharmacological effects of anti-inflammatory and anti-oxidation. Total glycoside of paeony could prevent diabetes-associated renal damage against oxidative stress via NF-kB p65 and p38 MAPK pathway (Jing et al., 2010). MC was found significantly increasing glutathione content and remarkedly decreasing induced nitric oxide synthase activity in hippocampus tissue.

Grey relational analysis (GRA) is an analytical method based on the development trend of the curve shape on each factor (Zhu et al., 2017). This method is generally employed to reveal the quantitative comparison of the development trend in a dynamic variation system (Abudukeremu et al., 2015). In this paper, GRA was carried out to study the relationship between the variation trend of chemical properties and anti-oxidative effects of MC. The compounds that most relative with anti-oxidation were screened out based on the results obtained from GRA, and could be considered as potent antioxidant candidates.

Network pharmacology is a new discipline based on the basic theories of systems biology. It conducts a comprehensive analysis of biological systems and further find the specific node with multiple targets (Zhu et al., 2018). This paradigm is capable of describing complex interactions among biological systems, drugs, and diseases from a network perspective and in this sense shares the holistic perspective of TCM (Ge et al., 2018). Network pharmacology has been increasingly applied to exploring the pharmacological mechanisms of medicinal plants and crude drugs (Chen et al., 2019; Zhang et al., 2019).

In previous study, we had established a quantitative HPLC-MS method and identified main constituents in MC (Hou et al., 2018). In the present study, we collected MCs from herbal market and quantified the main compounds and evaluated its antioxidant activities at first, at then the grey relational analysis was employed to predict the key components that acting on anti-oxidative activities based on the chemical contents and antioxidant activities. The uncovering antioxidant targets and pathways that the selected components involved were then revealed by network pharmacology and cell test verification.

## Materials and Methods

### Chemical Reagents

The standards (>98%, purity) of paeonol, paeoniflorin, benzoylpaeoniflorin, paeonolide, gallic acid and oxypaeoniflorin were purchased from Chengdu Herbpurify co, LTD. ABTS (2, 2′-azino-bis-(3-ethylbenzothiozoline-6-sulfonic acid) and DPPH (2, 2-diphenyl-1- picrylhydrazyl) were obtained from Sigma Aldrich, USA. HPLC grade acetonitrile was got from Tedia Company, Inc, USA (Fairfield, OH, United States). Formic acid with HPLC grade was obtained from Chongqing Chuandong Chemical co, Ltd. Methanol for extraction was purchased from Chendu Jinshan Chemical Reagent co, Ltd. Folin & Clocalteu’s phenol reagents were purchased from Shanghai Macklin Biochemical Co., Ltd.

### Sample Collection and Preparation

45 batches of MC samples were purchased from Chongqing herbal medicine market. The samples were from eight different production areas, in which 3 samples were collected from Guangxi province (GX1-3); 10 batches were from Chongqing (CQ1-10); 13 batches were from Anhui province (AH1-13); 10 batches were from Sichuan province (SC1-10); the other nine samples were from Henan province (HN1-3), Hubei (HB1-3), Shanxi (SXI) and Shandong province (SD1-2), respectively. The voucher samples were deposited at college of pharmaceutical sciences and Chinese medicine, Southwest University.

Duramens were removed from MC samples before be used. Then samples were pulverized and filtered through an 80-mesh sieve. 0.50 g dried powder sample was extracted with 50 ml 70% methanol aqueous solution for 30 min in an ultrasonic water bath (KQ5200E, 40 kHz). After extraction, add solvent to bring the volume to 50 ml. Then the extract was centrifuged at 10,000 rpm for 10 min, and the supernatant was collected and filtered through a 0.22 μm syringe filter before analysis. All samples were analyzed in triplicates.

### Quantification of Six Main Components in Moutan Cortex

In previous study, we found paeonol, paeoniflorin, benzoylpaeoniflorin, paeonolide, gallic acid and oxypaeoniflorin (Supplementary Figure S1) which were the predominant components in MC, and the quantitative analytical method with simultaneously quantifying the six ingredients in MC was established (Ge et al., 2019). Briefly, The HPLC analyses were performed using a LC-20A liquid chromatography system (Shimadzu Co., Japan) and samples were separated on an Ecosil C_18_ (250 mm × 4.6 mm, 5 μM, Lubex Co., China). The eluent solvents consist of the deionized water with 0.2% formic acid (A) and acetonitrile (B) using a gradient program of 0–3 min, 90–89.2% A; 3–5 min, 89.2–88% A; 5–9 min, 88–87.8% A; 9–10 min, 87.8–87.7% A; 10–15 min, 87.7–87% A; 15–18 min, 87–85% A; 18–21 min, 85–84.2% A; 21–26 min, 84.2–26% A; 26–30 min, 26–10% A; 30–35 min, 10% A. The elution was performed with the eluent solvent at a flow rate of 1.0 ml/min, and 10 µl of sample solution was injected into the LC system for analysis. Two ultraviolet spectra were monitored for acquiring chromatograms of six components, at 230 nm for paeoniflorin and benzoylpaeoniflorin and 274 nm for gallic acid, paeonol, paeonolide and oxypaeoniflorin, respectively. In this study, the established method was applied to analyze the contents of paeonol, paeoniflorin, benzoylpaeoniflorin, paeoniflorin, gallic acid and oxypaeoniflorin of MCs to reflect its chemical properties.

### Measurement of Antioxidant Activity by DPPH/ABTS^+^

The antioxidant activity of MC extracts was determined by using DPPH free radical scavenging assay with minor modification (Dai et al., 2013). Briefly, DPPH powder was dissolved in 100 ml methanol to freshly prepare the 0.05 mM DPPH solution for DPPH method. The prepared solution was stored at 4°C in dark. For each reaction, 0.1 ml of sample solution was mixed with 3.9 ml DPPH stock solution and incubated for 30 min in dark. As a negative control, 3.9 ml of DPPH solution and 0.1 ml of 70% methanol aqueous solution were used. Then the absorbance of reaction was measured at 517 nm using a UV spectrophotometer (METASH, UV-6100) with an ascorbic acid (Vc) comparison. Additional dilution was needed if the DPPH value measured was over the linear range of the standard curve. All above samples were run in six replicates. The scavenging activity (SC) of samples was expressed through the following formula:

SC (100%) =100% × (A0 - A1)/A0

Where A_0_ and A_1_ represent the absorbance of negative control and sample, respectively.

The scavenging activity of ABTS radical was determined according to the reported method with slight modifications (Re et al., 1999). The ABTS radical cation was prepared by the reacting ABTS with potassium persulphate. The mixture was incubated in dark at room temperature for 12 h. Then the ABTS radical cation solution was diluted with methanol to give an absorbance of 0.70 ± 0.05 at 734 nm. After adding 0.2 ml of the MC extract to 2.0 ml of diluted ABTS radical cation solution, the absorbance was recorded after 15 min incubation. The above mentioned samples were analyzed in six replicates.

### Grey Relational Analysis

The grey relational analysis (GRA) was utilized as an evaluation system to assess the effects of the diverse existing compounds on antioxidant activity in this study (Deng, 1989). The content of paeonol, paeoniflorin, benzoylpaeoniflorin, paeonolide, gallic acid and oxypaeoniflorin together with the scavenging activities from DPPH and ABTS method was selected to be a grey system. The specific GRA procedure is as follows:

Firstly, the raw data of associated factors are normalized and then the deviation sequences are determined. Finally, the grey relational coefficients are calculated by the following equations:

$$\mathrm{Set}\;x_{0}=\left(x_{0}\left(1\right),x_{0}\left(2\right),\ldots ,\;x_{0}\left(n\right)\right)$$

$$\;x_{1}=\;\left(\;x_{1}\left(\text{1}\right)\text{, }x_{1}\left(\text{2}\right)\text{, }...\text{, }x_{1}\left(\text{n}\right)\right)\text{;}$$

$$\;x_{2}=\;\left(\;x_{2}\left(\text{1}\right)\text{, }x_{2}\left(\text{2}\right)\text{, }...\text{, }x_{2}\left(\text{n}\right)\right)\text{;}$$

...

$$\;x_{\text{i}}\;=\;\left(x_{\text{ i }}\left(\text{1}\right)\text{, }x_{\text{i}}\;\left(\text{2}\right)\text{, }...\text{, }x_{\text{i }}\left(\text{n}\right)\right)$$

as the sequence of associated factors.

The correlation coefficient is defined as the following equation:

$$\gamma \left(x_{0}\left(k\right),x_{i}\left(k\right)\right)=\frac{\underset{i}{\min}\underset{k}{\min}|x_{0}\left(k\right)-x_{i}\left(k\right)\mathrm{|+}\xi \underset{i}{\max}\underset{k}{\max}|x_{0}\left(k\right)-x_{i}\left(k\right)|}{|x_{0}\left(k\right)-x_{i}\left(k\right)\mathrm{|+}\xi \underset{i}{\max}\underset{k}{\max}|x_{0}\left(k\right)-x_{i}\left(k\right)|}$$

Where ξ is the distinctive coefficient lying between 0 and 1, which is set as to be 0.1.

The grey relation grade (GRG) is formulated as follows:

$$\gamma \left(x_{0},x_{i}\right)=\frac{1}{n}\sum_{k=1}^{n}\gamma \left(x_{0}\left(\kappa \right),x_{i}\left(\kappa \right)\right)$$

Where *n* is the number of performance characteristics.

The influence degree of the factors including the contents of components and antioxidant activities on the research object was estimated by comparing their GRG value. The higher GRG value between two associated factors is, the closer sequence of the two factors would be.

### Network Pharmacology Study

In order to further recognize the mechanisms of the underlying the antioxidant effects on the targeted compounds from GRA, the network pharmacological approach was used, including the evaluation of the targeted compounds’ Absorption, Distribution, Metabolism, and Excretion (ADME) properties, prediction of compounds-related targets, and recognition of core functions and modules via the protein-protein interaction (PPI) network approach. We identified a core modulatory network and found the main pathway that involved in the antioxidant activities of the targeted compounds.

Firstly, the structurally similar drugs of the selected components of CM were screened by using MedChem studio, and then the antioxidant targets of these drugs were obtained from the DrugBank database and considered as the putative targets of the selected components of CM. Next, we collected all the known therapeutic targets of drugs contributed to antioxidant effects from DrugBank20 (http://www.drugbank.ca/, version 4.3) and the Online Mendelian Inheritance in Man (OMIM) database21 (http://www.omim.org/). After overlap analysis of these collected targets, the putative CM target-known therapeutic targets of the antioxidant network were constructed using the links between putative targets of CM and known antioxidant targets.

To further clarify the pathways involved in the putative CM targets, the pathway enrichment analysis was performed by using the database Visualization and Integrated Discovery software32 (DAVID, http://david.abcc.ncifcrf.gov/home.jsp, version 6.7) and based on the pathway data obtained from the Kyoto Encyclopedia of Genes and Genomes database (KEGG, http://www.genome.jp/kegg/).

### Cell Viability Assay

RAW 264.7 cells were incubated in DMEM medium supplemented with 10% FBS and 1% penicillin/streptomycin. The cells were cultured in 96-well plates and cultivated for 48 h while exposed to different treatments. Cell viability was measured by MTT method. Briefly, incubated with different compounds for 12 h, the cell culture was washed twice with warm PBS buffer, and then fresh medium with 200 μM t-BHP was added to the cell culture. After incubation for 1 h, the culture medium was replaced with fresh medium containing 5 mg/ml MTT and incubated another 4 h, and then the medium were removed and 100 µl of dimethyl sulfoxide (DMSO) was immediately added to the wells. The reagents were thoroughly mixed and assayed at 540 nm on a SYNERGY H1 microplate reader.

### ROS Levels in the Cells

The detection of intracellular ROS levels was conducted according to the reported methods (Hui et al., 2021). RAW 264.7 cells were seeded in plates with a density of 5,000 cells per well. After 48 h incubation, the cells were treated with the three components at 5, 10, 20, and 40 μM for 12 h, respectively and then the treated cells were washed and incubated with 10 μM of H2DCF-DA for 30 min. Extracellular H2DCF-DA was removed by washing the cultures twice with warm PBS. The cellular oxidative stress was induced by incubating the cells with 200 μM t-BHP in PBS for 1 h. The cellular fluorescence intensities of each well were measured and recorded with a SYNERGY H1 microplate reader. The excitation and emission filters were set at 485 and 535 nm, respectively. The results are expressed as the percentages between the inhibition of the fluorescence relative and the untreated controls. Values of fluorescence intensity were obtained from at least six independent samples for each compound tested.

### Gene Expression of TNF, ALB, VEGFA and Caspase3

Total RNA was extracted using a Qiagen RNeasy Mini kit (Qiagen, Inc, USA). and cDNA was synthesized using a PrimeScript™ RT reagent Kit (TaKaRa Inc, Japan). The reverse transcription-polymerase chain reaction (RT-PCR) was applied to evaluate the mRNA expression of TNF, ALB, VEGFA and Caspase3. RT-PCR primers for these genes were followed: sense (5′-CCC​TCA​CAC​TCA​CAA​ACC​AC-3′) and antisense (5′- CAC​CAC​AGG​GCA​AAG​GAG​AT-3′) for TNF; sense (5′-AA-GAC​GTG​TGT​TGC​CGA​TGA-3′) and antisense (5′-GGC​CTT​TCA​AAT​GGT​GG-CAG-3′) for ALB; sense (5′- GGG​AGT​CTG​TGC​TCT​GGG​AT-3′) and antisense (5′-GGT​GTC​TGT​CTG​TCT​GTC​CG-3′) for VEGFA; sense (5′-GGG​GAG​CTT​GGA​ACG​CTA​AG-3′) and antisense (5′- CCG​TAC​CAG​AGC​GAG​ATG​AC-3′) for Caspase3; sense (5′- TGC​TCC​TCC​CTG​TTC​CAG​AG-3′) and antisense (5′- CTC​GTG​GTT​CAC​ACC​CAT​CA-3′) for GAPDH. The following PCR conditions were applied at 95°C for 2 min, 40 cycles of 95°C for 15 s and 60°C for 1 min.

### Statistical Analysis

The experimental data were presented as means of six replicates determination ±standard deviation. All statistical analyses were carried out using a SPSS20 software, Graphpad Prism software or online software.

## Results

### Quantitative Analysis of Six Main Compounds in MC

As shown in Table 1, paeonol and paeoniflorin were the main ingredients of MC among the six detected components due to their contents accounting for more than 80% the whole detected components, furthermore paeonolide had the lowest content. The content of paeonol ranged from 13.85 to 26.08 mg/g and paeoniflorin also showed a great variety ranging from 3.95 to 14.31 mg/g. The results of quantitative analysis indicated that the contents of the main components had great variation among MCs from the herbal market. Furthermore, Hierarchical Cluster Analysis (HCA) was applied to discriminate MCs based on the contents of the six quantified components. The results showed that most samples could gather together except the samples of CQ1, AH12 and AH13 that formed a small branch (Supplementary Figure S2). The HCA results indicated the holistic chemical properties of MCs were relative stable.

**TABLE 1:** Contents of the six constituents in 45 batches of MC (mg^.^g^−1^, x±SD, *n =6*).

Samples	Gallic acid	Oxypaeoniflorin	Paeonolide	Paeoniflorin	Benzoyl-paeoniflorin	Paeonol
GX1	1.23 ± 0.10	1.58 ± 0.08	0.38 ± 0.07	7.10 ± 0.08	0.92 ± 0.05	18.81 ± 1.21
GX2	1.26 ± 0.06	1.64 ± 0.06	0.25 ± 0.06	6.44 ± 0.07	1.19 ± 0.04	21.76 ± 0.38
GX3	1.10 ± 0.08	1.49 ± 0.05	0.18 ± 0.04	7.81 ± 0.08	0.94 ± 0.04	15.27 ± 0.42
CQ1	0.36 ± 0.04	3.43 ± 0.08	0.24 ± 0.05	14.24 ± 0.09	0.64 ± 0.05	18.17 ± 0.53
CQ2	1.76 ± 0.07	0.78 ± 0.04	0.31 ± 0.04	3.95 ± 0.06	0.68 ± 0.06	18.63 ± 0.17
CQ3	0.82 ± 0.03	2.56 ± 0.09	0.23 ± 0.05	10.36 ± 0.07	1.07 ± 0.08	19.88 ± 0.12
CQ4	0.48 ± 0.06	2.63 ± 0.15	0.75 ± 0.07	14.31 ± 0.15	0.93 ± 0.07	17.74 ± 0.20
CQ5	1.04 ± 0.08	1.65 ± 0.07	0.32 ± 0.06	7.25 ± 0.07	0.99 ± 0.06	19.16 ± 1.25
CQ6	0.73 ± 0.05	1.45 ± 0.07	0.13 ± 0.08	7.48 ± 0.06	0.75 ± 0.07	16.07 ± 0.47
CQ7	1.25 ± 0.06	1.19 ± 0.06	0.22 ± 0.05	5.45 ± 0.08	0.73 ± 0.06	19.17 ± 0.26
CQ8	1.37 ± 0.05	1.64 ± 0.08	0.43 ± 0.07	8.03 ± 0.06	1.05 ± 0.05	21.67 ± 0.19
CQ9	0.67 ± 0.08	2.49 ± 0.04	0.57 ± 0.06	11.56 ± 0.07	0.76 ± 0.08	20.01 ± 0.27
CQ10	1.25 ± 0.06	1.41 ± 0.05	0.17 ± 0.08	7.54 ± 0.06	0.89 ± 0.07	16.56 ± 0.32
AH1	1.08 ± 0.06	1.43 ± 0.07	0.14 ± 0.05	5.83 ± 0.07	0.77 ± 0.06	17.67 ± 0.13
AH2	1.09 ± 0.05	1.54 ± 0.09	0.28 ± 0.05	6.85 ± 0.06	1.21 ± 0.05	18.39 ± 0.23
AH3	1.15 ± 0.07	1.59 ± 0.08	0.18 ± 0.06	7.06 ± 0.07	1.07 ± 0.08	15.79 ± 0.14
AH4	1.08 ± 0.08	1.76 ± 0.05	0.26 ± 0.06	8.54 ± 0.06	1.05 ± 0.08	17.55 ± 0.09
AH5	0.52 ± 0.05	1.38 ± 0.05	0.13 ± 0.08	5.92 ± 0.07	0.83 ± 0.05	17.22 ± 0.12
AH6	0.83 ± 0.06	1.56 ± 0.07	0.10 ± 0.06	7.95 ± 0.06	0.84 ± 0.04	17.34 ± 0.09
AH7	0.80 ± 0.04	1.43 ± 0.10	0.22 ± 0.07	6.20 ± 0.06	0.74 ± 0.06	16.59 ± 0.08
AH8	1.07 ± 0.08	1.24 ± 0.07	0.27 ± 0.08	6.53 ± 0.04	0.80 ± 0.05	17.96 ± 0.10
AH9	1.06 ± 0.07	1.79 ± 0.06	0.16 ± 0.05	8.05 ± 0.06	0.77 ± 0.07	21.74 ± 0.13
AH10	0.75 ± 0.05	1.38 ± 0.06	0.26 ± 0.07	6.93 ± 0.05	0.76 ± 0.07	17.38 ± 0.11
AH11	0.50 ± 0.03	1.87 ± 0.03	0.54 ± 0.07	10.96 ± 0.06	1.87 ± 0.09	19.98 ± 0.16
AH12	0.84 ± 0.06	2.15 ± 0.07	0.14 ± 0.04	10.71 ± 0.05	1.50 ± 0.05	26.08 ± 1.02
AH13	0.33 ± 0.06	3.09 ± 0.06	0.41 ± 0.09	11.47 ± 0.07	0.85 ± 0.08	18.56 ± 0.11
SC1	1.15 ± 0.07	1.48 ± 0.09	0.19 ± 0.04	7.78 ± 0.09	0.99 ± 0.07	14.55 ± 0.10
SC2	1.12 ± 0.04	1.46 ± 0.08	0.13 ± 0.06	7.64 ± 0.07	1.00 ± 0.06	15.69 ± 0.09
SC3	0.77 ± 0.07	1.40 ± 0.06	0.16 ± 0.08	7.50 ± 0.06	0.78 ± 0.04	16.75 ± 0.14
SC4	1.13 ± 0.08	1.70 ± 0.06	0.16 ± 0.05	7.38 ± 0.09	0.71 ± 0.05	17.74 ± 0.09
SC5	1.14 ± 0.07	1.13 ± 0.05	0.34 ± 0.07	6.33 ± 0.05	0.73 ± 0.09	17.49 ± 0.13
SC6	1.05 ± 0.04	1.70 ± 0.04	0.22 ± 0.01	8.49 ± 0.05	0.88 ± 0.07	18.59 ± 0.09
SC7	1.16 ± 0.04	1.51 ± 0.07	0.17 ± 0.07	8.03 ± 0.06	0.84 ± 0.06	16.63 ± 0.12
SC8	0.95 ± 0.06	1.46 ± 0.08	0.17 ± 0.08	7.25 ± 0.07	0.77 ± 0.06	15.30 ± 0.09
SC9	1.02 ± 0.07	1.92 ± 0.06	0.08 ± 0.04	8.10 ± 0.08	1.16 ± 0.08	22.83 ± 0.14
SC10	1.26 ± 0.08	1.40 ± 0.07	0.23 ± 0.08	7.55 ± 0.08	0.99 ± 0.04	19.92 ± 0.13
HN1	0.76 ± 0.06	1.36 ± 0.07	0.19 ± 0.09	7.27 ± 0.06	0.80 ± 0.07	15.75 ± 0.09
HN2	0.95 ± 0.05	2.25 ± 0.08	0.16 ± 0.08	9.71 ± 0.05	1.15 ± 0.06	21.11 ± 0.23
HN3	1.13 ± 0.07	2.14 ± 0.06	0.13 ± 0.05	9.65 ± 0.06	1.20 ± 0.04	23.45 ± 0.34
HB1	0.86 ± 0.04	1.74 ± 0.05	0.20 ± 0.05	7.64 ± 0.01	0.90 ± 0.07	14.86 ± 0.09
HB2	1.75 ± 0.07	1.73 ± 0.07	0.14 ± 0.07	7.35 ± 0.06	1.05 ± 0.08	13.85 ± 0.31
HB3	0.91 ± 0.05	1.13 ± 0.06	0.66 ± 0.08	5.78 ± 0.05	0.71 ± 0.06	16.28 ± 0.12
SX1	0.92 ± 0.07	1.57 ± 0.05	0.28 ± 0.07	7.75 ± 0.08	0.82 ± 0.05	18.99 ± 0.09
SD1	1.18 ± 0.07	1.52 ± 0.04	0.45 ± 0.05	7.36 ± 0.07	1.11 ± 0.06	14.96 ± 0.08
SD2	0.72 ± 0.05	1.31 ± 0.06	0.77 ± 0.06	7.55 ± 0.08	0.86 ± 0.07	14.38 ± 0.09

### Antioxidant Properties of MC Based on DPPH and ABTS Assays

**FIGURE 1:**
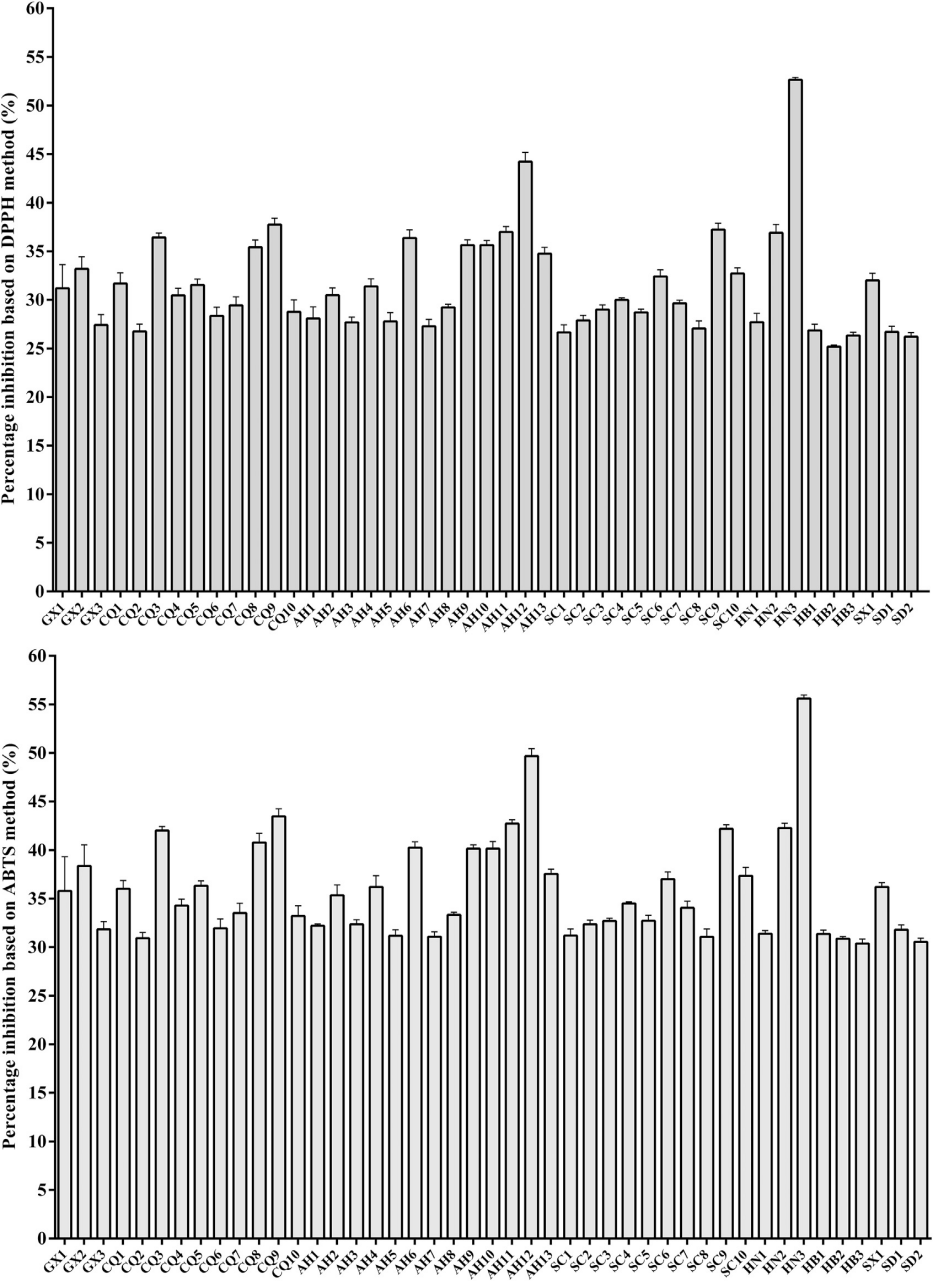
Effect of various MC extracts on the activity of DPPH and ABTS.

The antioxidant activity assays based on DPPH and ABTS free radical scavenging activities were applied in the present study (Figure 1). The results from antioxidant property assays showed that all MCs were capable of directly reacting with and quenching DPPH and ABTS radicals. The MC samples of HN3, AH12 and CQ9 had exhibited the potent antioxidant effects, while the samples of HB3, HB2 and SD2 showed the lower antioxidant activities. The data concluded from the two methods displayed a similar tendency of antioxidant activities of MCs. Besides, the MC samples were gathered into two groups by Hierarchical Cluster Analysis (HCA), in which AH12 and HN3 were clustered into one small group, while other samples were gathered into another big branch (Supplementary Figure S3). The HCA results from anti-oxidative activity assay were different from with that of chemical analysis.

**FIGURE 2:**
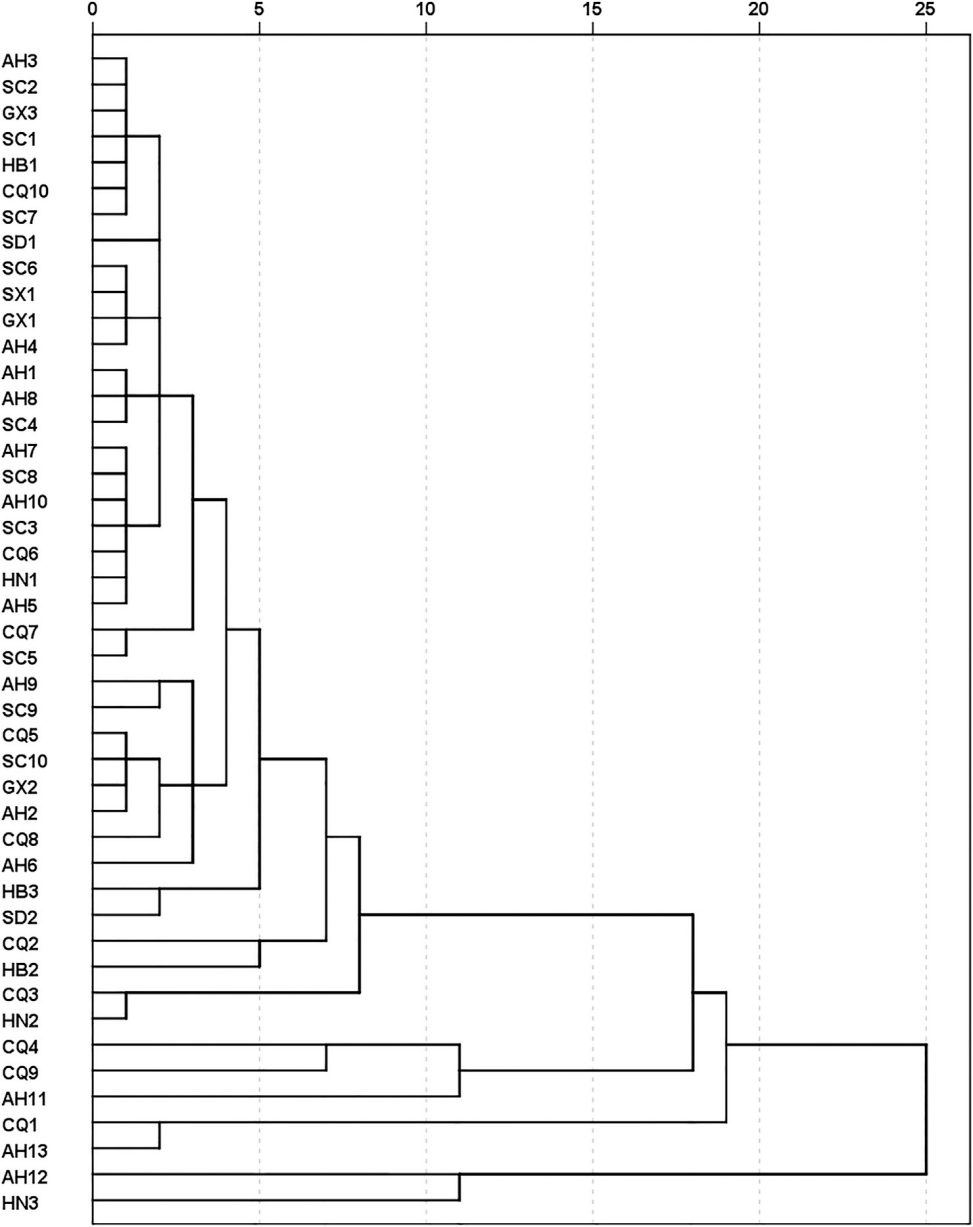
HCA dendrogram of the 45 batches of MC samples based on the combined data of contents of detected six components and anti-oxidative activities.

The experimental data was combined from the quantitative analysis and anti-oxidative activity assay and then was imported it into a SPSS statistics 20 software for further analysis. Firstly, the raw data was normalized and then was used for HCA. As shown in Figure 2, most samples were classified into one big group, while the sample AH12 and HN3 gathered into a small branch, indicating that the chemical and the anti-oxidative bioactivity properties of the 2 MC samples showed a great discrepancy from other MC samples. However, the samples planted from the same province always scattered among other samples and could not gather into a group, possibly resulting from the different processing procedure or species.

### Paeonol, Gallic Acid and Benzoylpaeoniflorin Were Evaluated as Potent Antioxidants by Grey Relational Analysis

The GRA was employed to predict the main antioxidant compounds of MC by comparing the tendency of anti-oxidative activity and contents of quantified ingredients among MCs. The average GRG were obtained and summarized in Table 2. The values of the GRG ranged from 0.6219 to 0.9584, implying that all the detected compounds had high influence on the anti-oxidative activity. According to GRG values from high to low, the orders of the components most related to the anti-oxidative activity were paeonol, gallic acid, benzoylpaeoniflorin, paeoniflorin, oxypaeoniflorin and paeonolide, respectively. So the components of paeonol, gallic acid and benzoylpaeoniflorin were thought as main factors contributing to anti-oxidative properties with high GRG above 0.62. The phenolic compound paeonol was eventually found possessing the highest anti-oxidative potentity with the GRG of 0.9584. Several relative literatures had reported that paeonol induced many pharmacological effects through inhibiting oxidative stress (Qin et al., 2010; Bao et al., 2013; Gong et al., 2017). As a member of a polyphenol family, gallic acid is considered to be one of the most abundant sources of nature antioxidants (Roidoung et al., 2016; Rajan and Muraleedharan, 2017; Ola-Davies and Olukole, 2018). Benzoylpaeoniflorin, as a monoterpene glycoside, also has been proved as a strong antioxidant from related studies (Fang et al., 2008; Xu et al., 2017). Therefore, the GRA results suggested that the components of paeonol, gallic acid, and benzoylpaeoniflorin could be considered as potential antioxidants of MC.

**TABLE 2:** Grey relational grade with rank order of six compounds detected in MCs.

Compounds	Average grey relational grade (*n* = 6)		Order
	DPPH	ABTS	
Gallic acid	0.8452 ± 0.092	0.8406 ± 0.061	2
Oxypaeoniflorin	0.7292 ± 0.032	0.7415 ± 0.055	5
Paeonolide	0.6219 ± 0.046	0.6287 ± 0.070	6
Paeoniflorin	0.7384 ± 0.078	0.7428 ± 0.042	4
Benzoylpaeoniflorin	0.7806 ± 0.084	0.7783 ± 0.076	3
Paeonol	0.9584 ± 0.036	0.9429 ± 0.069	1

### Network Pharmacology Analysis Predicted the Targets of Antioxidants

**FIGURE 3:**
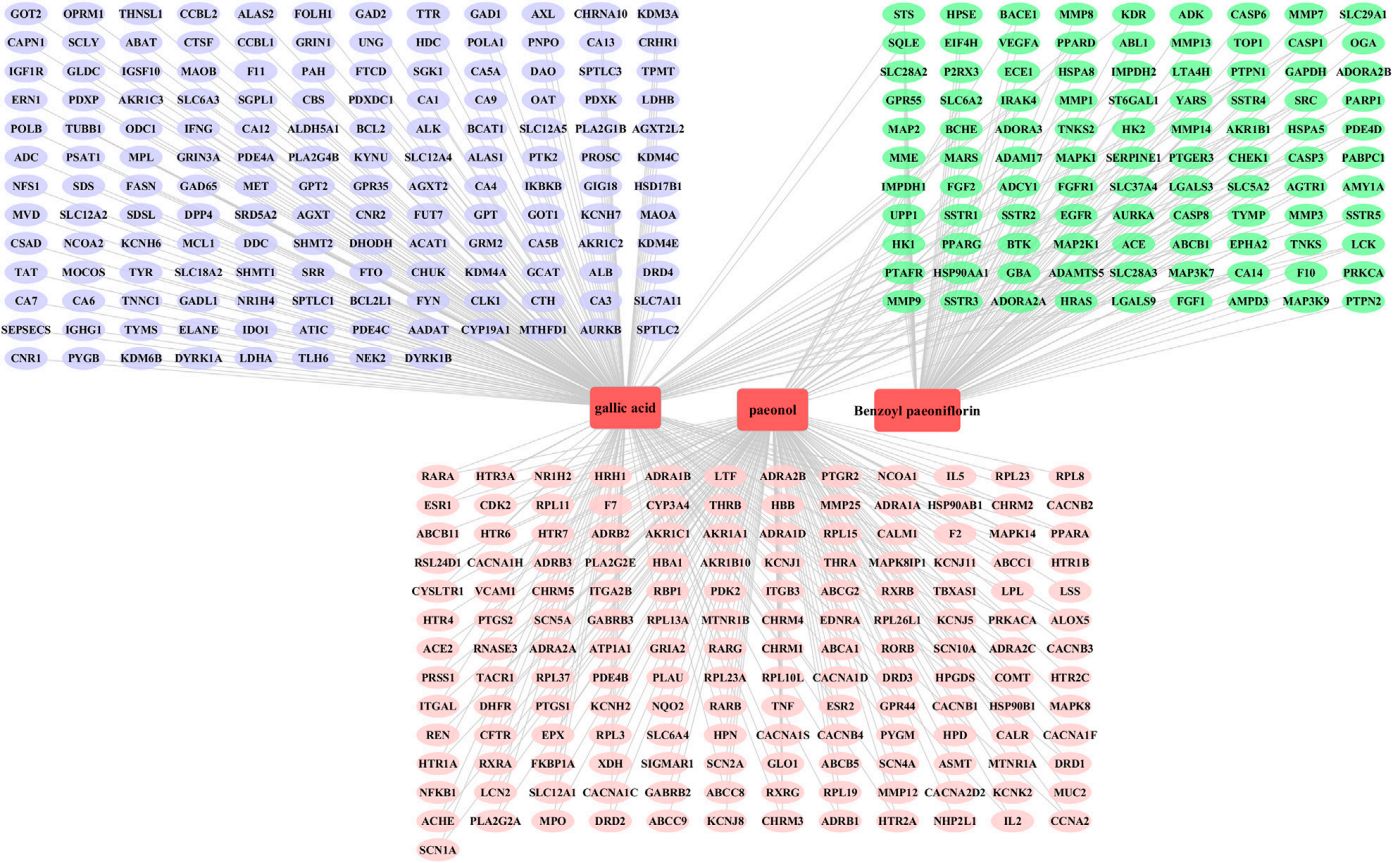
Compound-putative target network of gallic acid, benzoylpaeoniflorin and paeonol. Light blue circles, light green circles and light red circles represent the targets of compounds gallic acid, benzoylpaeoniflorin and paeonol, representatively. The yellow square frames represent the compounds.

To further uncover possible mechanism of the compounds (paeonol, gallic acid and benzoylpaeoniflorin) acting on anti-oxidative properties, we used network pharmacology strategy to predict the putative targets of these ingredients. First, 535 genes were selected and predicted as the putative targets of the three compounds, including 177 genes of paeonol, 258 genes of gallic acid and 100 genes of benzoylpaeoniflorin, and 408 targets were yielded after deletion of duplicates. Detailed information about the putative targets of the compounds was provided in Supplementary Table S2 and the compound-target network is shown in Figure 3. The analysis of component-target network included a total of 411 nodes and 533 edges, including three component nodes and 408 target nodes. The connecting components or the nodes with more target points play a pivotal role in the entire interaction network, which may be the key component or target gene that plays an antioxidant role in CMs.

Then the known therapeutic targets of drugs in the treatment of oxidation related diseases were collected. After removing redundant entries, 1,034 known therapeutic targets for the antioxidant activities were used for the further data analysis.

**FIGURE 4:**
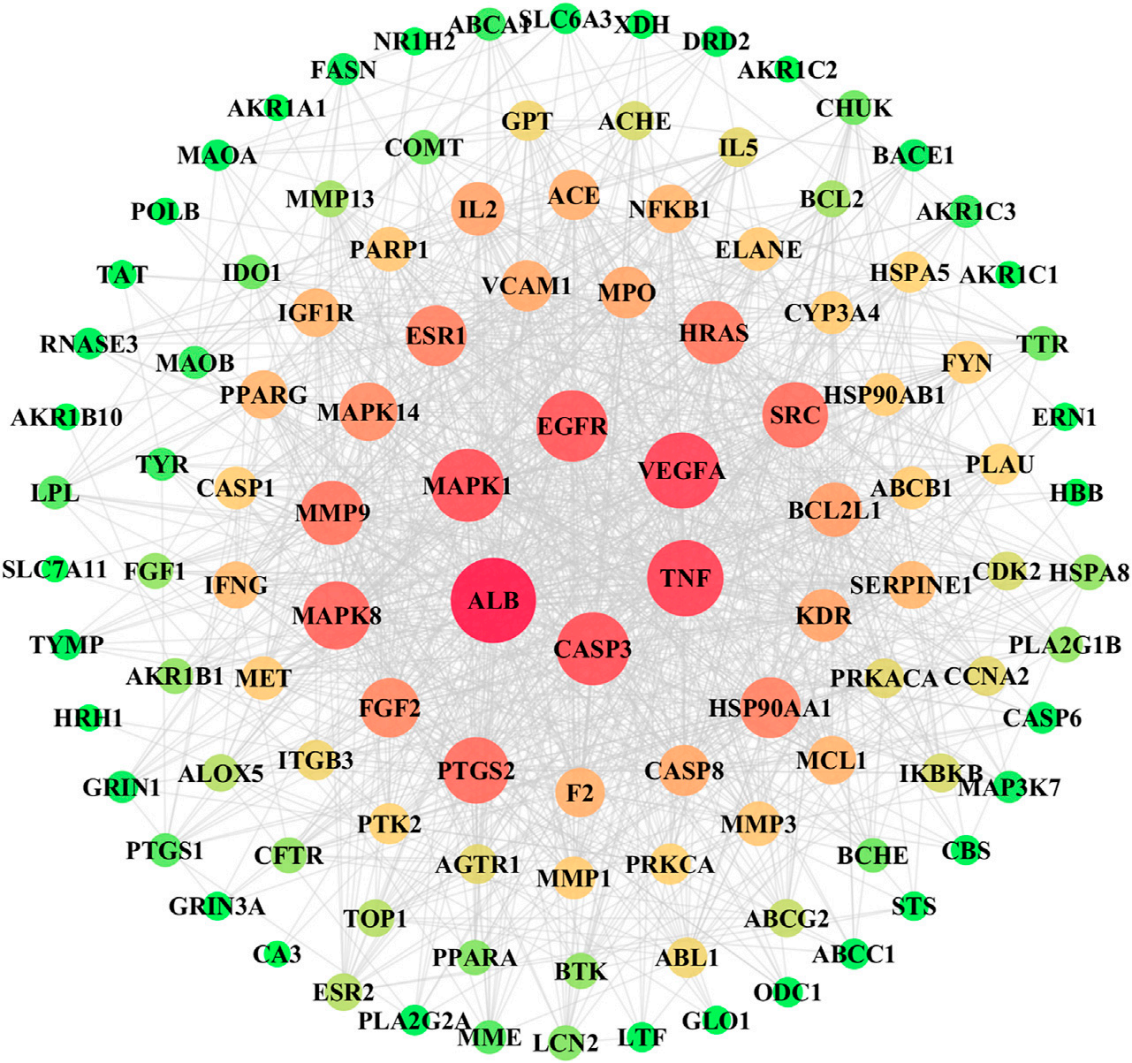
Clusters of the compound-disease target’ PPI network. The size and the color of the node represents the value of the degree, the thickness of the side indicates the value of the Combine score.

The known therapeutic targets of the antioxidant activity networks and putative compounds-target networks were then constructed. The interaction between the target proteins was shown in Figure 4, which included a total of 110 hubs, 1,184 edges, of which hubs represented the target protein and each edge represented the protein-protein interaction. In this network interaction, it has a network density of 0.197 with characteristics path length 1.966 and 21.527 average number of neighbours. The size and color of nodes was proportional with the degree. We found that albumin (ALB) (degree = 76), Tumor Necrosis Factor (TNF) (degree = 65), Vascular Endothelial Growth Factor A (VEGFA) (degree = 65), Caspase 3 (degree = 61), and Mitogen-Activated Protein Kinase 1 (MAPK1) (degree = 61) had more than 60° value and were centrally located in the protein-protein interaction network (PPI), indicating that these proteins were involved in the pathogenesis of oxidation.

**FIGURE 5:**
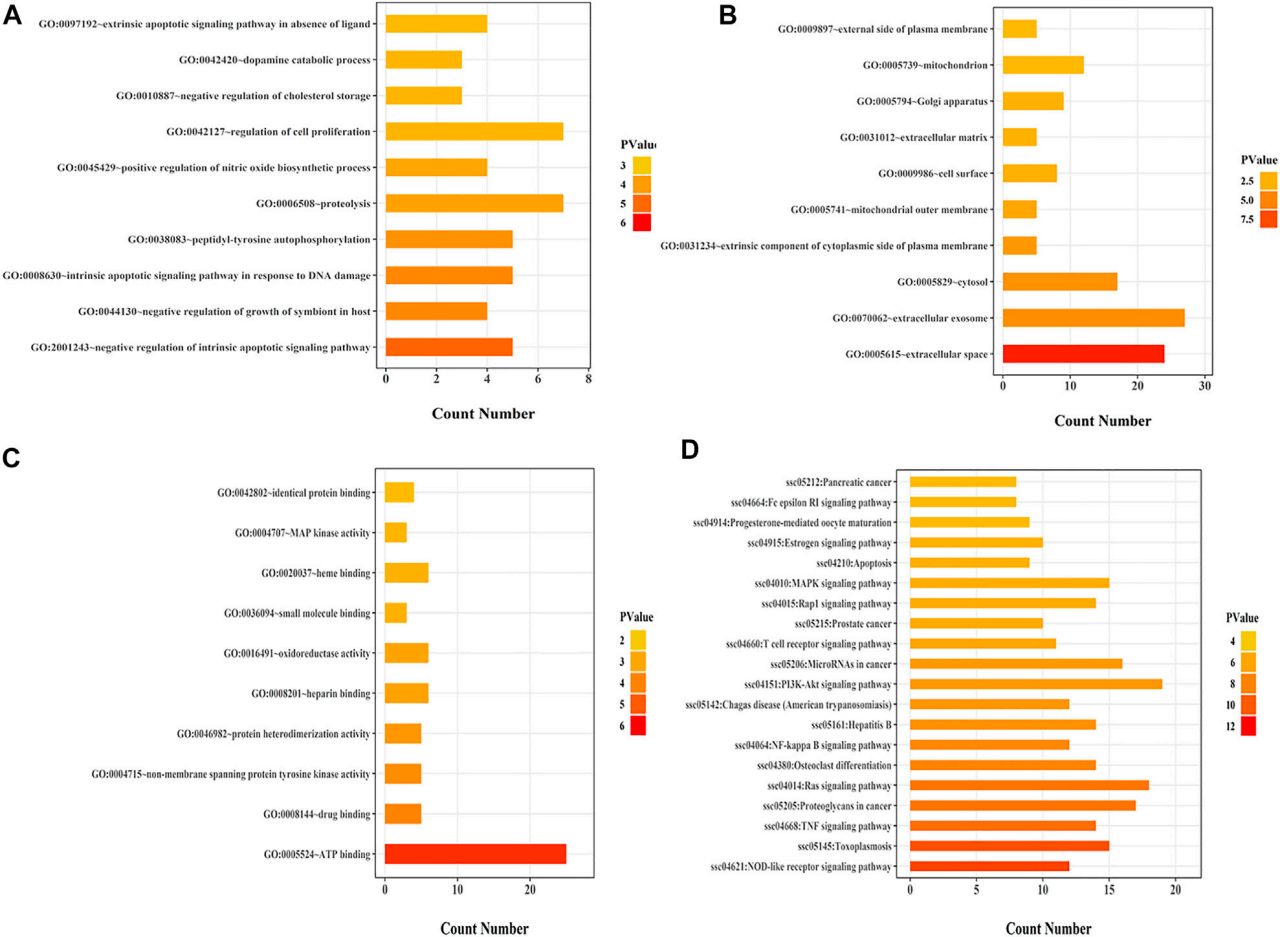
Target protein GO enrichment analysis and KEGG pathway analysis. **(A)**: The top ten significantly enriched terms in biological process (BP); **(B)**: The top ten significantly enriched terms in cellular component (CC); **(C)**: The top 10 significantly enriched terms in molecular function (MF); **(D)**: The top 10 significantly enriched terms in KEGG.

The predicted targets from PPI network mainly responded to many biological process, such as intrinsic apoptotic signaling regulation, DNA damage, peptidyl-tyrosine autophosphorylation, proteolysis, and nitric oxide biosynthetic process (Figure 5A). The cellular component analysis showed that the genes mainly related to extracellular exosome, cytosol, extrinsic component of cytoplasmic side of plasma membrane, mitochondrial outer membrane, cell surface, extracellular matrix, Golgi apparatus, mitochondrion and external side of plasma membrane (Figure 5B). There targets also involved in many protein receptor binding and protein activities, including ATP binding, drug binding, heparin binding, small molecule binding and non-membrane spanning protein tyrosine kinase activity, protein heterodimerization activity, oxidoreductase activity, MAP kinase activity (Figure 5C). To investigate the biological functions of these major hubs, a pathway enrichment analysis was performed. 107 KEGG pathways were obtained based on the PPI targets. As shown in Figure 5D, the major 10 KEGG pathways were significantly associated with various physiological processes, including NOD-like receptor signaling pathway, Toxoplasmosis, TNF signaling pathway, Proteoglycans in cancer, Ras signaling pathway, NF-kappa B signaling pathway and PI3K-Akt signaling pathway.

### Effects of Predicted Antioxidants on Cell Viability, ROS Levels and Gene Expressions of TNF, ALB, VEGFA, Caspase3 in t-BHP-stimulated RAW 264.7 Cells

**FIGURE 6:**
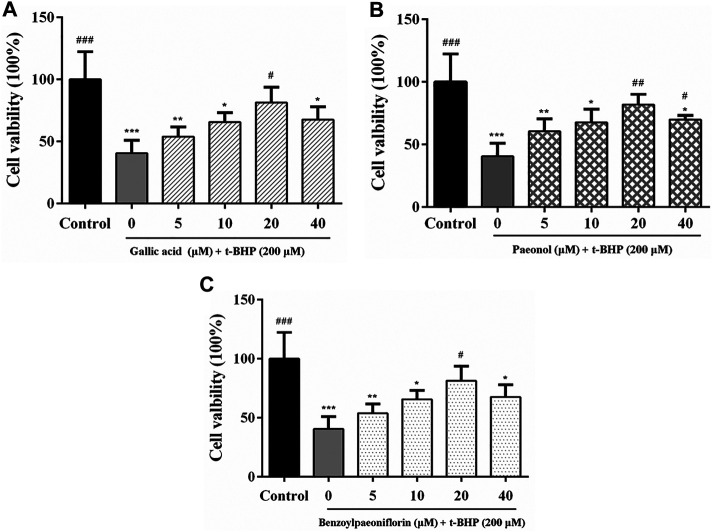
Evaluation of cell viability in RAW264.7 with the MTT assay. The components of gallic acid **(A)**, paeonol **(B)**, and benzoylpaeoniflorin **(C)** show protective effects in the t-BHP stimulated RAW264.7 cells. Data are given as mean ± standard deviation (*n* = 6). Compared with t-BHP treatment cell group, ###*p* < 0.001; ##*p* < 0.01; ##*p* < 0.01. Compared with Control, *** *p* < 0.001; ** *p* < 0.01; * *p* < 0.05.

**FIGURE 7:**
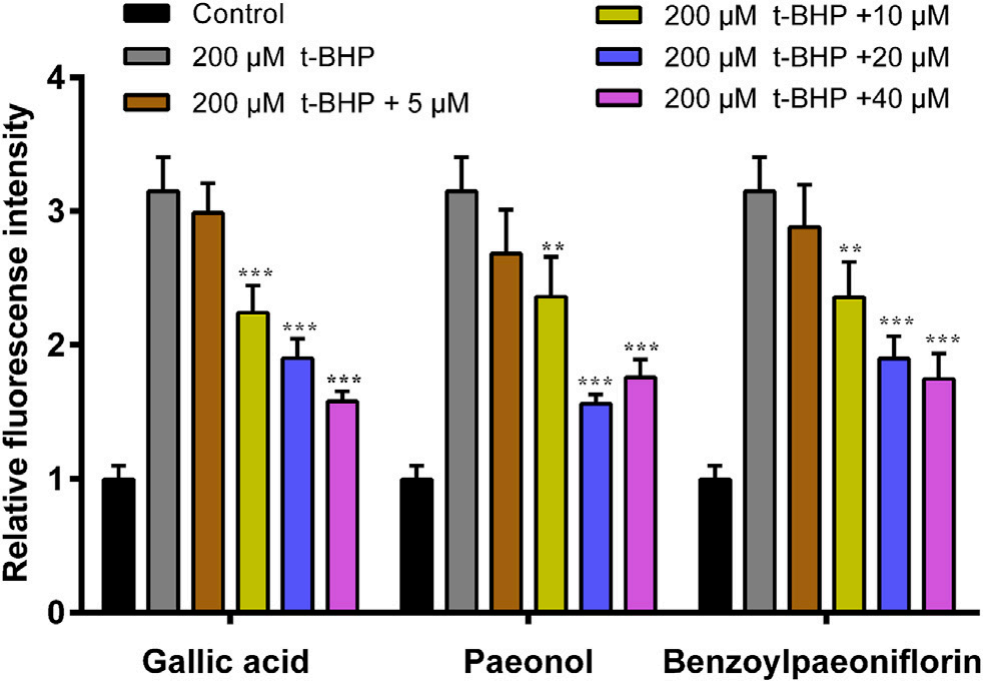
Modulation of excess ROS by gallic acid, paeonol and benzoylpaeoniflorin. Intracellular ROS was detected in t-BHP - induced RAW264.7 cells treated with indicated concentrations of gallic acid, paeonol and benzoylpaeoniflorin and quantified by DCFH2-DA fluorescence intensity assay. Compared with t-BHP treatment cell group, *** *p* < 0.001; ** *p* < 0.01; * *p* < 0.05.

A MTT assay was performed to evaluate the effects of the components on RAW264.7 cell viability. As shown in Figure 6, the cell viability of RAW 264.7 cells decreased to 40.5% after t-BHP stimulation, while the components of paeonol, gallic acid and benzoylpaeoniflorin could partly recover the t-BHP-stimulated cell viability and showed significantly difference at the concentration of 20 μM (*p* < 0.05). The t-BHP-induced intracellular ROS accumulation was monitored within cells using a DCFH2-DA fluorescence intensity analysis. The results from cellular ROS level further indicated that these components could clearly inhibit the generation of cellular ROS induced by t-BHP. Among the components, the treatment of gallic acid and benzoylpaeoniflorin showed a dose-effect relationship, and paeonol treatment presented the most ROS inhibition at the concentration of 20 μM (Figure 7).

**FIGURE 8:**
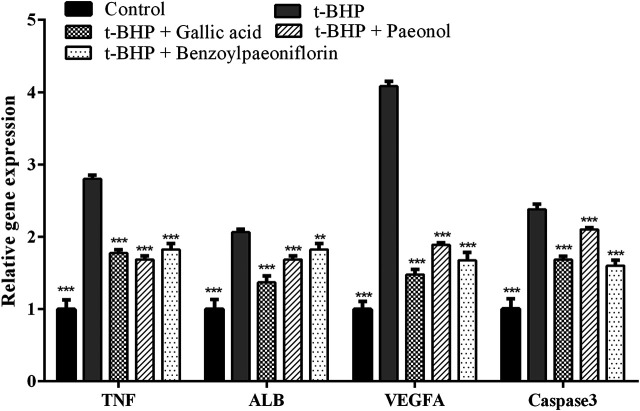
The expression patterns of target genes in RAW264.7 macrophages treated with gallic acid, paeonol and benzoylpaeoniflorin. The relative gene expressions were evaluated by qRT-PCR. Compared with t-BHP treatment cell group, *** p < 0.001; ** p < 0.01; * p < 0.05.

The genes of TNF, ALB, VEGFA and Caspase3 were predicted as the oxidative relative targets of the three components through network pharmacology analysis. Thus, we considered that the protective effects of the components against t-BHP-stimulated oxidative stress related to those target genes. The results from gene expression assay demonstrated that these ingredients could lead to the most significant decrease in the expression of the TNF, ALB, VEGFA and Caspase3 genes (Figure 8). This result is in accordance with the reduction of intracellular ROS in RAW264.7 cells. To sum up, these findings prove that the three bioactive compounds from MC could significantly prevent t-BHP-induced intracellular ROS generation in macrophages and protect the cells from apoptosis induced by oxidative stress (*p* < 0.001). Therefore, it can be suggested that the enrichment of paeonol, gallic acid and benzoylpaeoniflorin in MC could present as potential antioxidants.

## Discussion

Previous study had revealed that more than 100 compounds were isolated and identified from *Paeonia suffruti*cosa Andr, in which monoterpene glycosides and phenols were the predominant constituents (Wang et al., 2019). In addition, peaonol, paeoniflorin and their derivatives were the representative components of monoterpene glycosides and phenols (Zhou and Lv, 2008; Wang P. et al., 2017; Wang Z. Q. et al., 2017). In the present study, we found peaonol and paeoniflorin were the main components and account for 80% contents of the total detected components, which were in accordance with our previous study (Ge et al., 2019). However, the chemical properties of MCs from herbal market showed great diversities, in which the contents of peaonol and paeoniflorin ranged from 13.85 to 26.08 mg/g and 3.95–14.31 mg/g, respectively. Many factors may result in this variation. Several studies had demonstrated that the different origins of MCs showed great diversities on chemical properties (He et al., 2014). Besides, different cultivated areas and processing procedures also results in chemical diversities in MCs.

The components of paeonol, gallic acid and benzoylpaeoniflorin obtained high grades based on the chemical and bioactive evaluation by grey relational analysis and thus these compounds were predicted as key components of MC that acting on anti-oxidative activities. It is well known phenolic acids present strong antioxidant capacities (Akram et al., 2019), so there is no doubt that paeonol and gallic acid belonging to phenolic acid are selected as potent antioxidants. Benzoylpaeoniflorin is a derivative of paeoniflorin, and compounds with the same parent structure have shown therapeutic effect in experimental diabetic nephropathy by preventing diabetes-associated renal damage against oxidative stress (Fang et al., 2019). Besides, paeoniflorin and its derivatives had been found significant protection effects by ameliorating oxidative stress or involving a decrease in ROS production *in vivo* (Picerno et al., 2011; Zhao et al., 2013; Song et al., 2017).

Network pharmacology strategy is a potent tool to reveal the putative mechanisms of drugs like herbal medicine, which always are a complex chemical composition. In the present study, we had selected three key anti-oxidative components based on grey relational analysis. Thus, clarifying the mechanism of antioxidant action of these compounds by network pharmacology approach is a key imperative. Consequently, the compound-putative target network, PPI network with common targets for antioxidant, compound-disease were built to systematically analyze the mechanism of antioxidant action of the selected compounds. This network pharmacology study predicted the following four potential targets: ALB, TNF, VEGFA and Caspase3. Among these, ALB functions in the regulation of blood plasma colloid osmotic pressure and acts as a carrier protein for a wide range of endogenous molecules. Human serum albumin appears to reduce oxidative stress via NADPH oxidase inhibition in the human vascular smooth muscle, indicating that the serum level may be a critical determinant of vascular oxidative stress in some human diseases (Kinoshita et al., 2017). TNF is a cytokine secreted by macrophages, and involve in the regulation of a wide spectrum of biological functions. It has been reported that TNF-α/TNFR1 pathway involved in LPS alleviated APAP-induced oxidative stress (Zhao et al., 2019). Oxidative stress also had been found highly correlated with the presence of TNFA subgroup in patients with diabetes, diabetic nephropathy and chronic lymphocytic leukemia (Dabhi and Mistry, 2015; Jevtovic-Stoimenov et al., 2017). Caspases are a family of proteases involved in many important biological processes including apoptosis and inflammation. Antioxidants like sulfated corn bran polysaccharides could significantly inhibit the proliferation of A549 and HepG2 cell lines by the up-regulation at the mRNA expression level of pro-apoptotic genes Caspase3, Caspase8, Caspase9 (Xu et al., 2016). Panina et al. found hyperbaric oxygenation induced oxidative stress could significantly upregulate caspase-3-like activity and expression of Caspase3 mRNA in the cerebral cortex of rat, while addition of antioxidant led to the normalization of caspase3-like activity (Panina et al., 2018). The cell test in this study also further proved that the candidate genes closely related to reduction of oxidative stress, which was due to the treatment of the three components.

By KEGG enrichment analysis, the top 10 pathways were mainly related to cancer and immune system. As we know, one theory of tumorigenesis is from oxidative stress that activates inflammatory pathways leading to transformation of a normal cell to tumor cell. Oxidative stress can activate a variety of transcription factors including NF-κB, AP-1, p53, HIF-1α, PPAR-γ, β-catenin/Wnt, and Nrf2. Activation of these transcription factors can lead to the expression of over 500 different genes including inflammatory cytokines (Reuter et al., 2010). Extensive research has revealed the mechanism by which continued oxidative stress can lead to chronic inflammation, which in turn could mediate most chronic diseases including cancer (Bartsch and Nair, 2006; Grivennikov et al., 2010; Grivennikov and Karin, 2010).

## Conclusion

Taken together, the present results revealed that MC had significant antioxidant activity, and the compounds of paeonol, gallic acid and benzoylpaeoniflorin could be considered as promising antioxidant candidates of MC and markers for quality control of MC. Furthermore, a comprehensive method based on chemical analysis, bioactivity activity assays coupled with grey relational analysis was established to identify antioxidant candidates from MC, and network pharmacology strategy was proved to be an efficient tool for uncovering pharmacological mechanism of active ingredients.

## Data Availability

The datasets presented in this study can be found in online repositories. The names of the repository/repositories and accession number(s) can be found in the article/Supplementary Material.
