# Supplementary figures and images for: Grey Relational Analysis Combined With Network Pharmacology to Identify Antioxidant Components and Uncover Its Mechanism From Moutan Cortex
Source: Front Pharmacol. 2021 Oct 7;12:748501. doi: 10.3389/fphar.2021.748501 (PMC8529071; doi:10.3389/fphar.2021.748501)

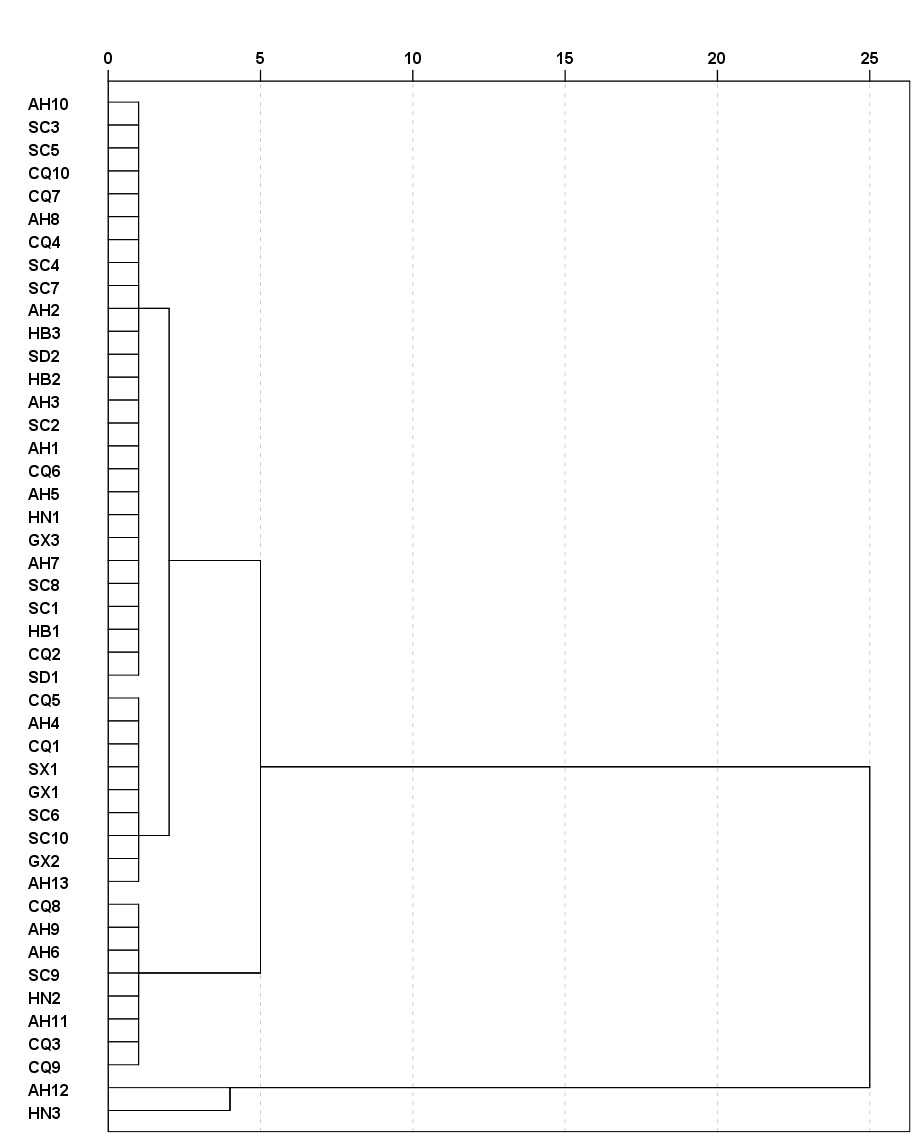

Supplement: Supplementary file 3 [file Image2.TIF]

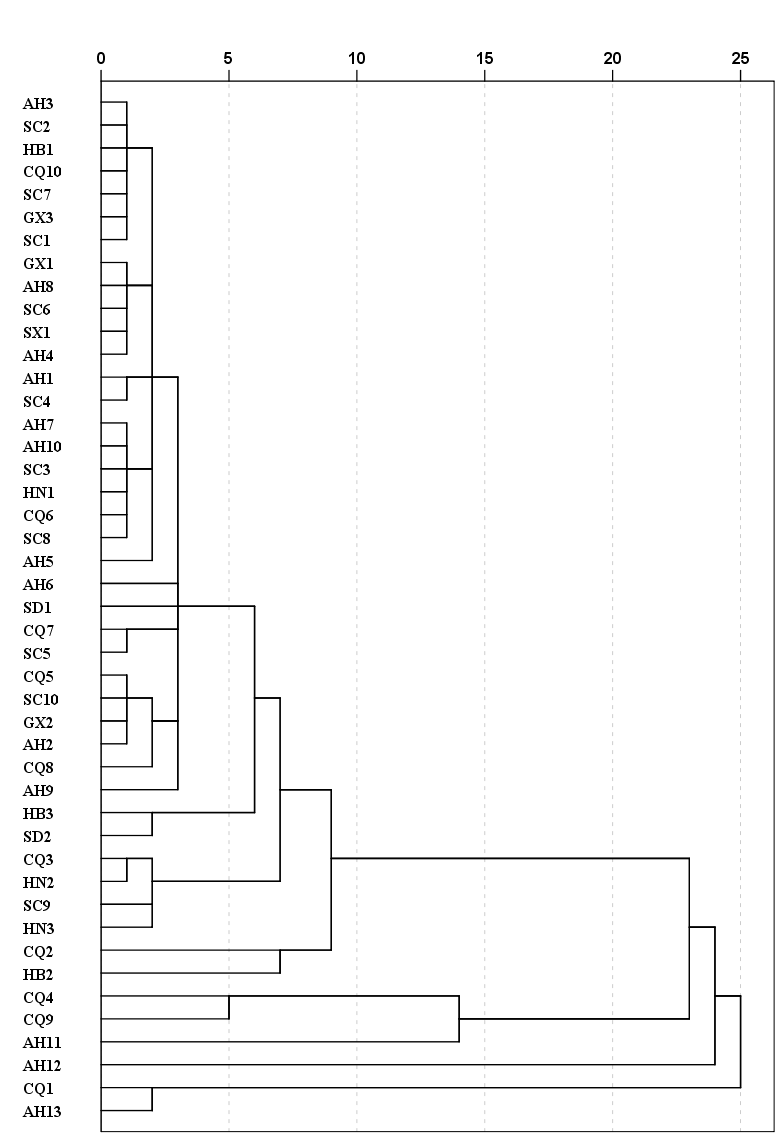

Supplement: Supplementary file 4 [file Image1.TIF]

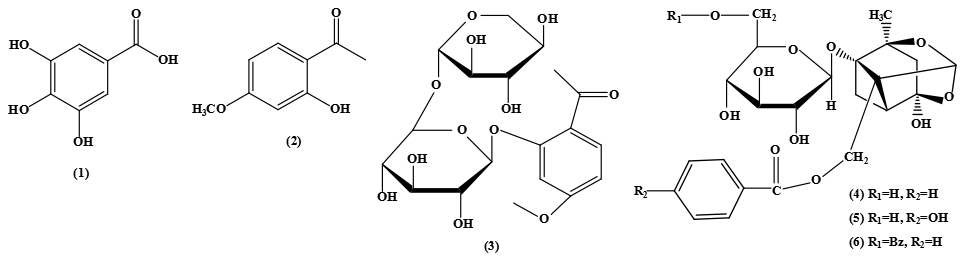

Supplement: Supplementary file 5 [file Image4.PNG]
